# The F-Box Protein Fbp1 Regulates Virulence of *Cryptococcus neoformans* Through the Putative Zinc-Binding Protein Zbp1

**DOI:** 10.3389/fcimb.2021.794661

**Published:** 2021-12-27

**Authors:** Lian-Tao Han, Yu-Juan Wu, Tong-Bao Liu

**Affiliations:** ^1^ State Key Laboratory of Silkworm Genomic Biology, Southwest University, Chongqing, China; ^2^ Chongqing Key Laboratory of Microsporidia Infection and Control, Southwest University, Chongqing, China; ^3^ Medical Research Institute, Southwest University, Chongqing, China

**Keywords:** *Cryptococcus neoformans*, ubiquitin-proteasome system, Fbp1, virulence, zinc-binding protein

## Abstract

The ubiquitin-proteasome system (UPS) is the major protein turnover mechanism that plays an important role in regulating various cellular functions. F-box proteins are the key proteins of the UPS, responsible for the specific recognition and ubiquitination of downstream targets. Our previous studies showed that the F-box protein Fbp1 plays an essential role in the virulence of *C. neoformans*. However, the molecular mechanism of Fbp1 regulating the virulence of *C. neoformans* is still unclear. In this study, we analyzed the potential Fbp1 substrates using an iTRAQ-based proteomic approach and identified the zinc-binding protein Zbp1 as a substrate of Fbp1. Protein interaction and stability assays showed that Zbp1 interacts with Fbp1 and is a downstream target of Fbp1. Ubiquitination analysis *in vivo* showed that the ubiquitination of Zbp1 is dependent on Fbp1 in *C. neoformans*. Subcellular localization analysis revealed that the Zbp1 protein was localized in the nucleus of *C. neoformans* cells. In addition, both deletion and overexpression of the *ZBP1* gene led to the reduced capsule size, while overexpression has a more significant impact on capsule size reduction. Fungal virulence assays showed that although the *zbp1*Δ mutants are virulent, virulence was significantly attenuated in the *ZBP1* overexpression strains. Fungal load assay showed that the fungal burdens recovered from the mouse lungs decreased gradually after infection, while no yeast cells were recovered from the brains and spleens of the mice infected by *ZBP1* overexpression strains. Thus, our results revealed a new determinant of fungal virulence involving the post-translational regulation of a zinc-binding protein.

## Introduction

*Cryptococcus neoformans* is an encapsulated yeast pathogen that can cause fatal fungal meningitis in immunocompromised individuals (Chayakulkeeree and Perfect, 2006; Pukkila-Worley and Mylonakis, 2008). In recent decades, with the increasing number of immunocompromised people such as AIDS patients or those who have received organ transplants and immunosuppressive therapy (Brown et al., 2012), the morbidity and mortality of cryptococcal infection has also increased significantly (Kronstad et al., 2011; Erwig and Gow, 2016). According to the latest estimates, *Cryptococcus* causes at least 250,000 infections and at least 181,000 deaths per year (Rajasingham et al., 2017). Despite the high morbidity and mortality from cryptococcosis, treatment options for cryptococcosis are limited, and only three major groups of drugs are currently approved for clinical use: polyenes (e.g., Amphotericin B), azoles (e.g., Fluconazole), and the pyrimidine analogue flucytosine (5-FC) (Day et al., 2013; May et al., 2016). However, the fact that the efficacy of these therapeutics is hampered by host toxicity and pathogen resistance also adds to the public health burden (Iyer et al., 2021). Therefore, Denning and Bromley have commented that the major barrier of fungal treatment is that our general lack of capacity in fungal pathogen research hinders our understanding of fungal diseases and the development of faster diagnostic methods and new therapies (Denning and Bromley, 2015). Given the shortage of anti-fungal drugs and the objective facts of fungal drug resistance, the pathogenesis of *C. neoformans* and the mechanism of drug resistance have become a hot and challenging spot that remains to be explored.

The ubiquitin-proteasome system (UPS) is the major pathway of intracellular protein degradation, consisting of an E1 ubiquitin-activating enzyme, an E2 ubiquitin-conjugating enzyme, and an E3 ubiquitin ligase, responsible for the degradation of more than 80% of proteins in the cell (Glickman and Ciechanover, 2002; Lecker et al., 2006; Deshaies and Joazeiro, 2009). The E3 ubiquitin ligases are the most critical enzymes in the UPS, responsible for the specific recognition and ubiquitination of substrates (Ardley and Robinson, 2005). The E3 ubiquitin ligases are generally divided into two broad structural classes: the HECT (homologous to the E6AP carboxyl terminus) domain family and the RING (really interesting new gene) domain family (Ardley and Robinson, 2005). The SCF (Skp1, Cullins, and F-box proteins) E3 ubiquitin ligases are among the best-understood groups of the RING ligases. The SCF E3 ligases have been linked to various human diseases, such as neurodegenerative disorders and cancer (Ardley and Robinson, 2004; Devoy et al., 2005), and SCF E3 ligase-mediated UPS has emerged as an attractive drug target for human diseases (Sakamoto, 2002; Mitsiades et al., 2005).

F-box proteins are proteins containing an F-box domain consisting of about 50 amino acids, which is the protein structural motif initially identified in human cyclin F (Bai et al., 1994; Bai et al., 1996). The F-box protein interacts with the Skp1 of the SCF E3 ligase through the F-box domain at its N terminus and binds to the substrate through its C-terminal protein-binding domain, thereby specifically identifying the substrate for ubiquitination and degradation (Jonkers and Rep, 2009). F-box proteins exist in all eukaryotic organisms and have a large variety of functions. So far, many F-box proteins have been identified in fungi, and these proteins play an essential role in regulating cellular functions such as cell cycle, circadian clocks, nutrient sensing, and signal transduction (Jonkers and Rep, 2009). The first fungal F-box protein identified and well-studied was the glucose repression resistant gene *GRR1* in *S. cerevisiae* (Flick and Johnston, 1991; Jonkers and Rep, 2009). Studies have revealed that Grr1 is involved in cell cycle regulation, nutrient sensing, and fungal morphogenesis by regulating its downstream targets (Flick and Johnston, 1991; Bai et al., 1996; Bernard and Andre, 2001; Blondel et al., 2005). The function of Grr1 homologs in other fungi has also been reported, including the Grr1 in *Candida albicans* (Butler et al., 2006), the GrrA in *Aspergillus nidulans* (Krappmann et al., 2006), and the Fbp1 in *Gibberella zeae* (Han et al., 2007). Another F-box protein, Cdc4, has also been studied in *S. cerevisiae* (Feldman et al., 1997; Orlicky et al., 2003; Liu et al., 2011a) and *C. albicans* (Atir-Lande et al., 2005; Shieh et al., 2005; Tseng et al., 2010). Recent studies also showed that the F-box proteins are linked to the virulence of plant-pathogenic fungi (Duyvesteijn et al., 2005; Han et al., 2007; Shi et al., 2019; Lim and Lee, 2020). So far, there are also a few reports on the role of the F-box proteins in virulence in human fungal pathogens, such as Fbp1 in *C. neoformans* (Liu et al., 2011b) and Fbx15 in *Aspergillus fumigatus* (Johnk et al., 2016). However, besides *S. cerevisiae*, substrates of the F-box proteins in many fungi remain to be identified. Therefore, identification and functional study of the F-box proteins and their substrates in human fungal pathogens may help to improve our understanding of fungal virulence and may lead to novel approaches for controlling fungal infections.

In our previous studies, we identified an F-box protein Fbp1 that is essential for fungal virulence in *C. neoformans*. Further, we identified inositol phosphosphingolipid-phospholipase C1 (Isc1), a substrate of Fbp1 required for full fungal virulence, using co-immunoprecipitation (CoIP) and liquid chromatography-tandem mass spectrometry (LC-MS/MS) methods (Liu et al., 2011b; Liu and Xue, 2014). In this study, we systematically analyzed the potential substrates of Fbp1 using an iTRAQ-based proteomic approach and identified the zinc-binding protein Zbp1 as a substrate of Fbp1. Functional analysis revealed that the proper regulation of Zbp1 is required for full fungal virulence in *C. neoformans*. Thus, our results revealed a new determinant of fungal virulence involving the post-translational regulation of zinc-binding proteins.

## Materials and Methods

### Strains, Media, and Growth Conditions

The *C. neoformans* and *Saccharomyces cerevisiae* strains used in this study are listed in **Table S1**. The cryptococcal strains were routinely grown at 30°C on YPD agar or liquid medium. Strains containing *CTR4* promoter-controlled genes were grown on a YPD medium with the addition of 25 µM CuSO_4_ and 1 mM ascorbic acid or 200 µM bathoproproinediulonic acid (BCS) (Ory et al., 2004). The yeast strain Y187 was used for the yeast two-hybrid assay, and the transformants were grown on SD medium lacking leucine, tryptophan, histidine, or adenine. The J774 murine macrophage cells used for the *Cryptococcus*-macrophage interaction assay were grown in liquid Dulbecco modified Eagle’s medium (DMEM) as described previously (Liu et al., 2011b; Liu and Xue, 2014). All other media were prepared as described previously (Liu et al., 2011b).

### Protein Extraction and iTRAQ Analysis

*Cryptococcus* cells from three wild-type and three *fbp1*Δ mutant groups were grown in YPD broth overnight and harvested, respectively. Protein extracts were prepared as described previously (Bahn et al., 2005; Liu and Xue, 2014). Briefly, the harvested cells were washed twice with ice-cold double distilled water and transferred into a 2-mL bead-beating tube containing 600-µL acid-washed glass beads. The proteins were then extracted in lysis buffer (50 mM Tris-HCl, pH 7.4, 150 mM NaCl, 1 mM EDTA,1%Triton X-100) by lysing the cells at 4°C with glass beads (eight times for 40 s each) in a FastPrep-24 5G apparatus (MP Biomedical, USA). The protein extracts were collected after centrifugation at 14,000 rpm at 4°C for 20 min.

To explore the differentially expressed proteins following the deletion of the *FBP1* gene, we sent the total proteins of the wild-type strain H99 and the *fbp1*Δ mutant to Shanghai Applied Protein Technology Co., Ltd. (APTBIO, China) for iTRAQ coupled with LC-MS/MS analysis using the protocols described by Liu et al. and Wang et al. with minor modification (Liu et al., 2017; Wang et al., 2017). Briefly, 200 µg proteins for samples of the wild type and the *fbp1*Δ mutant were dissolved with 0.5 M triethylammonium bicarbonate after removing the detergent, DTT, or other low-molecular-weight components using UA buffer (8 M Urea, 150 mM Tris-HCl pH 8.0) by repeated ultrafiltration. The protein suspensions of each sample were digested with 4 µg trypsin (Promega) in 40 μL dissolution buffer overnight at 37°C. Then 100 µg peptide mixture of the wild type or the *fbp1*Δ mutant samples were labeled with iTRAQ reagents 115 and 116 (Applied Biosystems), respectively. The iTRAQ labeled peptides were pooled and purified by a strong cation exchange (SCX) chromatography using the AKTA Purifier system (GE Healthcare) and separated by liquid chromatography (LC) using an EASY-nLC 1200 HPLC system (Thermo Scientific). The LC fractions were analyzed using a Q Exactive mass spectrometer (Thermo Scientific) that was coupled to Easy nLC for 240 min. Briefly, the peptide mixture was separated using a nanoViper C18 column (Thermo Scientific) with a linear gradient of buffer (84% acetonitrile and 0.1% Formic acid) at a flow rate of 300 nL/min controlled by IntelliFlow technology. The mass spectrometer was operated in positive ion mode, and the MS data were acquired using a data-dependent top10 method with a mass range of 300-1800 m/z. iTRAQ data from three biological replicates were analyzed using the MASCOT engine (Matrix Science, London, UK; version 2.3.02), and the protein identification was performed using the most recently updated *C. neoformans* Uniprot database (Uniprot_Cryptococcus_neoformans_21752_20170103.fasta, 21752 sequences). The search parameters used to identify proteins were the same as described by Liu et al. (2017). The false discovery rate (FDR) was calculated using the PSPEP (Proteomics System Performance Evaluation Pipeline Software) algorithm with an automatic decoy database search strategy. Only proteins with at least one unique peptide and unused value of more than 1.3 were considered for further analysis. All data were reported on the basis of 99% confidence for protein identification with FDR of ≤ 1%. To calculate the relative protein levels, proteins with a statistically significant label ratio of ≥1.2 (*P* < 0.05) were considered to be differentially expressed proteins.

### Yeast Two-Hybrid Assays

To test the interaction between Fbp1 and Zbp1 proteins, we performed a yeast two-hybrid interaction assay as previously described (Osman, 2004; Liu et al., 2011b). The full-length cDNAs of *FBP1* and *ZBP1* were cloned into the bait vector pGBKT7 and fused with the BD domain. The cDNAs of *FBP1* and *ZBP1* were also cloned into the prey vector pGADT7 and fused with the AD domain. All inserted cDNA sequences were confirmed by sequencing. Both bait constructs and prey constructs were cotransformed into the yeast strain Y187. The transformation mixtures were plated on an SD medium lacking leucine and tryptophan (SD-Leu-Trp) at 30°C for 2-3 days. The transformants growing on an SD medium lacking histidine or adenine were considered positive interactions. Transformants containing the pGADT7-T7 and pGBKT7-53 served as positive controls, while transformants containing pGADT7-T7 and pGBKT7-LAM served as negative controls (Clontech, Dalian, China).

### Generation of Tagged Protein Strains

The cryptococcal strain expressing the Fbp1-Flag protein (TBL81) is from a prior study (Liu and Xue, 2014). To generate the strains expressing Zbp1-hemagglutinin (HA) proteins, we first amplified the cDNA of *ZBP1*with primers TL1252/TL892 and cloned them into the *Bam*HI sites of the pCTR4-2 vector (Ory et al., 2004) using an In-Fusion HD cloning kit (Clontech, Dalian, China), generating the Zbp1:HA fusion plasmid pTBL199. To test the interaction between Fbp1 and Zbp1 in *Cryptococcus*, we transformed the pTBL199 into the Fbp1-Flag strain biolistically, generating the strain TBL363 expressing both Fbp1:Flag and Zbp1:HA proteins. Total proteins were extracted and analyzed by immunoblotting with anti-Flag and anti-HA antibodies. Proteins were pulled down using SureBeads™ anti-Flag or anti-HA Magnetic Beads (Bio-RAD) and then analyzed by immunoblotting with anti-Flag to detect Fbp1-Flag or anti-HA antibodies to detect Zbp1-HA.

To test the stability of Zbp1 in the wild-type and *fbp1*Δ mutant strain backgrounds, we transformed the pTBL199 into the wild-type strain and an *fbp1*Δ mutant using a biolistic transformation to generate the Zbp1:HA-tagged strains TBL449 and TBL343, respectively. Then the strains TBL449 and TBL343 were grown to mid-logarithmic phase in YPD, transferred to YPD with 25 µM CuSO_4_ and 1 mM ascorbic acid, and further incubated for 1, 2, 4, and 6 h. Protein extracts were prepared as described above. Zbp1:HA was detected by Western blotting using a monoclonal anti-HA antibody (Sigma).

### Subcellular Localization Analysis of Zbp1

To determine the subcellular localization of Zbp1 in *C. neoformans*, we amplified the coding sequence of the *ZBP1* gene from the wild-type genomic DNA with primers TL1168/TL1169 and cloned into pCN19 to generate the GFP-Zbp1 fusion protein expression vector pTBL187. Then the resulting vector, pTBL187, was linearized with *Hind*III and biolistically transformed into the wild-type H99 or KN99**a** as described previously (Davidson et al., 2000). Stable transformants were selected on a YPD medium containing nourseothricin sulfate (100 mg/L). The fluorescence of the transformants was visualized using a confocal microscope (Olympus, FV1200). To visualize the nuclei of cryptococcal cells, we stained the yeast cells with a DAPI staining as described previously (Bahn et al., 2005; Liu et al., 2011b).

### Quantitative Real-Time PCR

To test the expression of the *ZBP1* gene under different conditions, we measured the *ZBP1* expression at mRNA levels under the condition of growing in YPD medium *via* quantitative real-time PCR (qRT-PCR). Yeast cells of each cryptococcal strain from the overnight culture were collected and washed with distilled H_2_O (dH_2_O). Following the manufacturer’s instructions, total RNA extraction and purification were performed using an Omega total RNA kit II (Omega Bio-tek, USA). Purified RNAs were quantified using a Nanodrop spectrometer (DeNovix, USA). The first-strand cDNA synthesis was performed using a Hifair^®^ II 1st Strand cDNA Synthesis Kit (Yeasen, Shanghai, China), following the manufacturer’s instructions.

Expression of *ZBP1* and *GAPDH* was analyzed using FastStart Essential DNA Green Master (Roche). Gene expression levels were normalized using the endogenous control gene *GAPDH*, and the relative levels were determined using the comparative threshold cycle (C_T_) method (Livak and Schmittgen, 2001). Quantitative real-time PCRs (qRT-PCRs) were performed using a LightCycler^®^96 QPCR system (Roche) as previously described (Xue et al., 2010).

### Generation of *zbp1*Δ Mutants and *ZBP1* Overexpression Strains

The *zbp1*Δ mutants were generated in both H99 and KN99**a** strain backgrounds using a split marker strategy as described previously (Kim et al., 2009; Fan et al., 2019). Briefly, the upstream and downstream flanking sequences of the *ZBP1* gene were amplified from H99 genomic DNA with primers TL1052/TL1053 and TL1054/TL1055, respectively (see **Table S2** in the **Supplementary Material** for primer sequences). The dominant selectable markers (*NEO*) were amplified with the M13 primers (TL17 and TL18) from plasmid pJAF1 (Fraser et al., 2003). Then the fusion fragments of the upstream flanking sequence and the 5′-region of the *NEO* marker were amplified by overlap PCR with primers TL1052/TL20 using the mixture of upstream flanking sequence and *NEO* marker as templates. The fusion fragments of the 5′-region of the *NEO* marker and the downstream flanking sequence were also obtained in the same way using primers TL19/TL1054. The two fusion fragments were combined and precipitated onto the gold microcarrier beads (0.6 µm; Bio-Rad) and biolistically transformed into the H99 and KN99**a** strains as described previously (Davidson et al., 2000). Stable transformants were selected on a YPD medium containing G418 (200 mg/L). The *zbp1*Δ mutants were further confirmed by diagnostic PCR using positive primers F4/R4(TL1058 and TL59) and negative primers F3/R3(TL1056 and TL1057, see **Table S2**) and Southern blot analysis.

To generate the complemented strain of the *zbp1*Δ mutant, we amplified a genomic DNA fragment containing a 1.5-Kb upstream promoter region, the *ZBP1* open reading frame (ORF), and its 0.5-Kb downstream region using primers TL1283/TL1284. This PCR fragment was cloned into the plasmid pTBL1, which contains the *NAT* selective marker gene to generate the complementation plasmid pTBL215. The pTBL215 was linearized by *Apa*I and biolistically transformed in both α and a mating-type *zbp1*Δ mutant strains.

To overexpress the *ZBP1* gene in *C. neoformans*, we amplified the *ZBP1* gene with primers TL1094/TL1095 and cloned it into the plasmid pTBL5 to generate pTBL175. The pTBL175 plasmid linearized with *Sal*I was biolistically transformed into the α mating-type *zbp1*Δ mutants. Stable transformants were selected on a YPD medium containing nourseothricin sulfate (100 mg/L). The overexpression of *ZBP*1 was confirmed by quantitative real-time PCR.

### Analysis of Melanin and Capsule Production

The Zbp1-related strains and the wild-type H99 strain were grown overnight in YPD broth at 30 °C. 100 μL of the culture of each strain was spotted on Niger seed agar medium to examine melanin production. The agar plates were incubated at 30°C or 37°C for 2 days, and the pigmentation of each strain was assessed and photographed. To examine capsule production, we washed the overnight culture of each strain three times with 1 × PBS buffer and incubated it in diluted Sabouraud medium or MM with mannitol overnight at 37°C (Zaragoza and Casadevall, 2004; Guimaraes et al., 2010), or in DME medium and incubated overnight in the presence of 5% CO_2_ (Liu et al., 2011b). The capsule size was quantified as described previously (Liu et al., 2011b).

### Virulence Studies

Overnight cultures of each *Cryptococcus* strain grown in YPD medium at 30°C were washed twice with PBS buffer and resuspended at a final concentration of 2 × 10^6^ cells/mL. Groups of ten female C57 BL/6 mice were anesthetized and intranasally inoculated with 1×10^5^ cells in 50 µL PBS of each cryptococcal strain as described previously (Cox et al., 2000). Throughout the experiments, animals were monitored and sacrificed by CO_2_ inhalation when they appeared moribund or in pain. Survival data from the murine experiments were statistically analyzed between paired groups using the log-rank test with PRISM version 8.0 (GraphPad Software, San Diego, CA) (A *P*-values < 0.05 were considered statistically significant).

### Histopathology and Fungal Burdens in Infected Tissues

Infected animals were sacrificed at the endpoint of the experiment or designated time points according to the Southwest University Institutional Animal Care and Use Committee (IACUC)-approved animal protocol. Infected brains, lungs, and spleens were collected, fixed in 10% formalin solution, and sent to the Servicebio biological laboratory for section preparation (Servicebio, Wuhan, China). Tissue slides were stained with hematoxylin and eosin (H&E) and examined by light microscopy. Meanwhile, infected tissues were also isolated and homogenized in 1× PBS buffer using a homogenizer. Resuspensions were diluted, and 100 µL of each dilution was plated on YPD agar with ampicillin and chloramphenicol. The numbers of colonies were counted after 2-3 days of incubation at 30°C to determine the fungal burdens at the time of sacrifice. All statistical analyses were performed by nonparametric Mann-Whitney test with PRISM version 8.0. P values of ≤0.05 were considered statistically significant.

### *Cryptococcus*-Macrophage Interaction Assay

A *Cryptococcus*-macrophage interaction assay was performed in 48-well culture plates, using J774 macrophage-like cells cultured in DMEM with 10% heat-inactivated FBS at 37°C with 5% CO_2_. A total of 5 × 10^4^ J774 cells in 0.5 mL fresh DMEM were added to each well of a 48-well culture plate and incubated at 37°C in 5% CO_2_ overnight. To activate macrophage cells, 50 units/mL gamma interferon (IFN-γ; Sigma) and 1 µg/mL lipopolysaccharide (LPS; Sigma) was added to each well. Overnight cultures of each *Cryptococcus* strain were washed with 1 ×PBS buffer twice and opsonized with 20% mouse complement (S3269; Sigma). A total of 2 ×10^5^ *Cryptococcus* cells were then added to each well for a 4:1 ratio of yeast to J774 cells. After 2 h of coincubation, nonadherent extracellular yeast cells were removed by washing with fresh DMEM, and cultures were incubated for another 0, 2, or 22 h to assess intracellular proliferation of *C. neoformans*. Distilled water (dH_2_O) was used at the indicated time points to replace the DMEM in each well and lyse the macrophage cells for 30 min at room temperature. The lysate was plated on YPD agar, and the CFU counts were used to determine intracellular proliferation.

### Serum Treatment Assay

To examine the cell viability of *Cryptococcus* in the presence of serum, we performed a serum treatment assay on the Zbp1-related strains. Overnight cultures of each strain were washed twice with distilled water (dH_2_O) and then resuspended in 450 µL of fetal bovine serum (FBS; Gibco) to a final concentration of 1 ×10^6^ cells/mL. The mixture of yeast cells and serum was incubated at 37 °C for 1, 2, 3, and 4 h. Then the aliquots were taken out at the indicated time points and spread onto the YPD medium after 10-fold serial dilution. The CFU counts were used to determine the cell viability of each *Cryptococcus* strain.

## Results

### Identification of the Zinc-Binding Protein Zbp1

As a key component of the E3 ligases, the F-box protein Fbp1 usually interacts with the phosphorylated substrates for ubiquitination and eventual degradation. To identify the downstream substrates of *Cryptococcus* Fbp1, we performed an iTRAQ coupled with LC-MS/MS analysis to explore the differentially expressed proteins in the *fbp1*Δ mutant backgrounds. By this approach, a total of 4387 proteins were detected and quantified (**Table S3**). A quantitative ratio over 1.2 (fold change > 1.2 or < 0.83) and a *P*-value < 0.05 were considered differentially expressed proteins. Using the criterion, we identified 105 proteins that were significantly upregulated, and 215 proteins were downregulated in *fbp1*Δ mutants (**Table S3**). Some proteins with higher abundance are shown in **Table 1**. These proteins are selected as potential substrate candidates for further investigation.

**Table 1:** Partial high abundance proteins identified in the *fbp1*Δ mutants of *C. neoformans*.

Accession	Description	Average* fbp1*Δ/H99	No. of PEST Domain
CNAG_05514	Uncharacterized protein	1.899037691	0
CNAG_05633	Ubiquinol-cytochrome c reductase subunit 9	1.527864633	0
CNAG_06871	Uncharacterized protein	1.458608795	1
CNAG_00576	Mitochondrial protein	1.381835321	0
CNAG_05350	Zinc-binding protein	1.381305021	2
CNAG_05216	CAMK protein kinase	1.378940229	1
CNAG_04669	Mitochondrial matrix protein import protein	1.370162857	0
CNAG_00582	Vacuolar transporter chaperone 1	1.369598183	0
CNAG_05817	GDP-mannose transporter 1	1.358720216	0
CNAG_06837	PH domain-containing protein	1.355891304	2
CNAG_05173	DNA-3-methyladenine glycosylase II	1.343807756	0
CNAG_02823	Bloom syndrome protein	1.307827849	2

**Figure 1:**
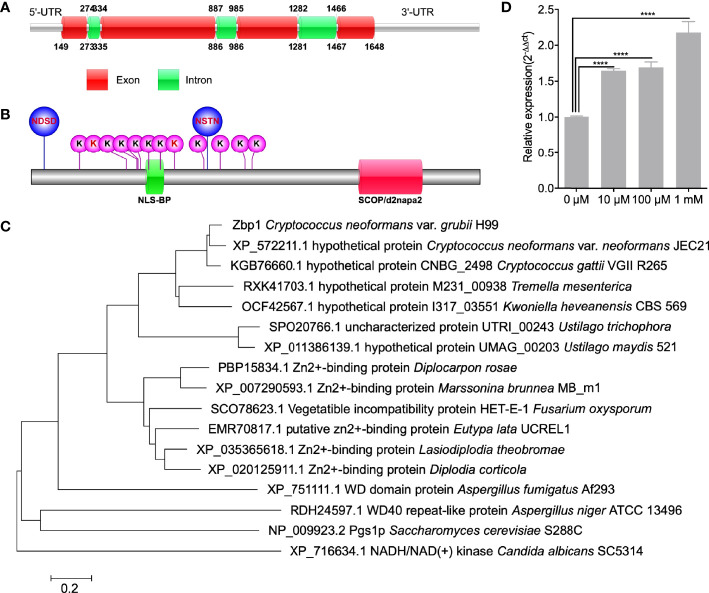
Identification of Zbp1 in *C. neoformans*. Schematic illustration of the *ZBP1* gene **(A)** and Zbp1 protein **(B)** in *C. neoformans*. NDSD and NSTN: Glycosylation site; K: Ubiquitination sites; NLS_BP: Bipartite nuclear localization signal profile; SCOP/d2napa2: SCOP d2napa2 domain. **(C)** Neighbor-joining phylogenic analysis of the Zbp1 (CNAG_05350) with its homologs in *Cryptococcus neoformans* var. *neoformans* JEC21 (XP_572211.1), *Cryptococcus gattii* (KGB76660.1), *Tremella mesenterica* (RXK41703.1), *Kwoniella heveanensis* (OCF42567.1), *Ustilago trichophora* (SPO20766.1), *Ustilago maydis* (XP_011386139.1), *Diplocarpon rosae* (BP15834.1), *Marssonina brunnea* (XP_007290593.1), *Fusarium oxysporum* (SCO78623.1), *Eutypa lata* (EMR70817.1), *Lasiodiplodia theobromae* (XP_035365618.1), *Diplodia corticola* (XP_020125911.1), *Aspergillus fumigatus* (XP_751111.1), *Aspergillus niger* (RDH24597.1), *Saccharomyces cerevisiae* (NP_009923.2), and *Candida albicans* (XP_716634.1). The bar marker indicates the genetic distance, which is proportional to the number of amino acid substitutions. **(D)** The expression of the *ZBP1* gene under different zinc ion concentrations. ****P < 0.0001.

One of the candidates, CNAG_05350, was highly abundant in the *fbp1*Δ mutant and contained two PEST domains in its sequence, indicating that CNAG_05350 is likely a downstream substrate of Fbp1, but this needs additional functional analysis for confirmation. Bioinformatic analysis from the FungiDB database (Basenko et al., 2018) indicates that the CNAG_05350 gene is 1500 bp in length, contains six exons, and encodes a 384 amino acid protein (**Figure 1A**). Protein sequence analysis showed that the CNAG_05350 protein has one bipartite nuclear localization signal profile (NLS_BP), one SCOP d2napa2 domain, two N-linked glycosylation sites (NGS), and twelve ubiquitination sites (**Figure 1B**). Sequence blast and phylogenetic analysis revealed that Zbp1 shows sequence similarity to a homolog of many species of basidiomycetes and ascomycetes (**Figure 1C**). However, most of the proteins from other species with high similarity to Zbp1 are hypothesized proteins whose functions are still unknown (**Figure 1C**). Sequence blast analysis also showed that CNAG_05350 protein has a 33-41% sequence similarity to the zinc-binding proteins of pathogenic fungi such as *L. theobromae*, *D. rosae*, *M. brunnea*, and *D. corticola* in **Table 2**, suggesting that the CNAG_05350 protein might be a zinc-binding protein (**Table 2**). We, therefore, name the CNAG_05350 protein zinc-binding protein (Zbp1) in *C. neoformans*. Since Zbp1 may be a zinc-binding protein and may involve in zinc homeostasis in *C. neoformans*, we first tested the expression of the *ZBP1* gene of the wild-type strain H99 under different zinc ion concentrations using Q-RT PCR. Our results showed that the expression of the *ZBP1* gene increased significantly (*P* < 0.0001) with the increase of zinc ion concentration, indicating that the Zbp1 protein may be a zinc ion binding protein (**Figure 1D**). Given the importance of zinc ion binding proteins in organisms, we decided to investigate the function of Zbp1 in *C. neoformans*.

**Table 2:** Comparison between Zbp1 and its homologs in other species.

Organism	Protein	Expect	Identities	Positives
*Lasiodiplodia theobromae*	Zinc-binding protein	6e-41	35%	45%
*Diplocarpon rosae*	Zinc-binding protein	6e-37	33%	45%
*Marssonina brunnea*	Zinc-binding protein	2e-34	45%	56%
*Diplodia corticola*	Zinc-binding protein	6e-30	41%	54%
*Fusarium oxysporum*	vegetatible incompatibility het-E-1	5e-35	31%	45%
*Aspergillus niger*	WD40 repeat-like protein	0.39	29%	47%
*Aspergillus fumigatus*	WD40 repeat-like protein	0.68	45.3%	56.6%
*Candida albicans*	Predicted NAD+/NADH kinase	0.89	30%	45%
*Saccharomyces cerevisiae*	Phosphatidylglycerolphosphate synthase, PGS1	0.21	21%	36%

### Zbp1 Interacts With Fbp1

**Figure 2:**
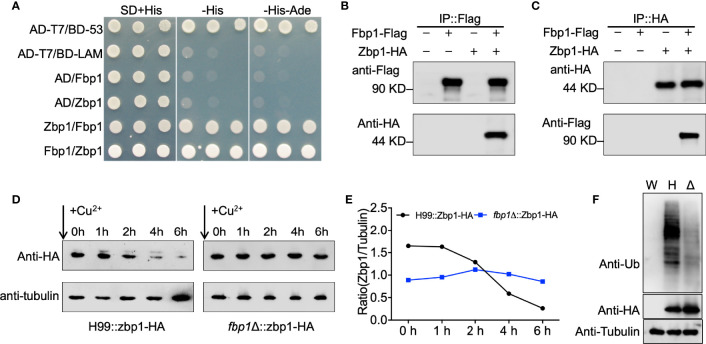
Zbp1 interacts with Fbp1 and is a substrate of Fbp1. **(A)** Zbp1 interacts with Fbp1 in a yeast two-hybrid interaction assay. The full length of the cDNAs of *ZBP1* and *FBP1* were fused with both the AD of vector pGADT7 and the BD of vector pGBKT7. Both fusion constructs were introduced into the yeast host strain Y187, and colonies grown on SD medium without leucine and tryptophan were tested on media also lacking histidine and lacking both histidine and adenine. Yeast cells expressing AD-T7 and BD-LAM or pGADT7 and pGBKT7 empty vectors were used as negative controls, while yeast cells expressing AD-T7 and BD-53 fusion proteins were used as positive controls. **(B, C)** Zbp1 interacts with Fbp1. Proteins from cryptococcal cells expressing Fbp1-Flag, Zbp1-HA, or both Fbp1-Flag and Zbp1-HA were extracted and purified. The potential Fbp1-Zbp1 interaction was analyzed by Co-IP with anti-Flag **(B)** or anti-HA antibody **(C)** and evaluated by immunoblotting. **(D)** The stability of the Zbp1 protein is dependent on the expression of Fbp1. The *P_CTR4_*-*ZBP1*:*HA* construct was expressed in the wild-type (H99) or *fbp1*Δ mutant background. Cells were grown in YPD to the mid-logarithmic phase. Cells were harvested after CuSO_4_ addition to stop *ZBP1* transcription at the indicated times, and the abundance of Zbp1 was monitored by immunoblotting using HA antibody. The expression of the tubulin gene was used as a loading control. **(E)** The data show the ratio of the band intensity of each Zbp1 protein to the corresponding tubulin at the indicated times. **(F)** Proteins from cryptococcal cells of the wild type (W), the wild type (H), or the *fbp1*Δ mutant (Δ) expressing Zbp1-HA were extracted and purified with HA antibody. The ubiquitination of Zbp1-HA was evaluated by immunoblotting with ubiquitin antibody. The expression of Zbp1-HA under the wild type or *fbp1*Δ mutant backgrounds was confirmed by immunoblotting with HA antibody. The expression of the tubulin gene was used as a loading control.

Our proteomics data showed that the Zbp1 protein is accumulated in the *fbp1*Δ mutant background and could be a substrate for Fbp1-mediated ubiquitination (**Table 1**). A substrate of Fbp1 will likely be accumulated in an *fbp1*Δ mutant background due to a lack of proper ubiquitination and degradation. To test the potential interaction between Zbp1 and Fbp1, we performed a yeast two-hybrid protein-protein interaction assay. Constructs containing Zbp1 and Fbp1 fused with either the activation domain (AD), or the binding domain (BD) were cotransformed into the yeast strain Y187, and interacting transformants were screened on SD-Leu-Trp-His and SD-Leu-Trp-His-Ade media. Based on this analysis, Zbp1interacted with Fbp1 (**Figure 2A**).

To further examine the interaction between Zbp1 and Fbp1, we generated a Zbp1:HA fusion expression construct in the vector pCTR4-2 in which Zbp1 was controlled by a copper-repressible *CTR4* promoter (Ory et al., 2004). The Zbp1:HA fusion protein expression construct was introduced into a previously generated Fbp1:Flag overexpression strain (Liu and Xue, 2014). The total proteins from the strain expressing Fbp1:Flag and Zbp1:HA were purified using SureBeads™ anti-Flag or anti-HA Magnetic Beads (Bio-RAD) and immunoblotted with anti-Flag and anti-HA antibodies. Both Flag and HA signals were detected from the Co-IP product, demonstrating that Zbp1 interacts with Fbp1 in *C. neoformans* (**Figures 2B, C**).

### The Stability of Zbp1 Is Dependent on Fbp1 Function

To evaluate the hypothesis that Zbp1 is a substrate of Fbp1, we examined whether the stability of the Zbp1 protein depends on the function of SCF(Fbp1) E3 ligase. The Zbp1:HA fusion construct was expressed in both the wild type and the *fbp1*Δ mutant, and the stability of the Zbp1:HA protein was examined in the background of these two strains. The strains that express the Zbp1:HA fusion protein were first cultured in a YPD medium containing 200 µM BCS to release the repression of the *CTR4* promoter by copper. The cultures were then washed with PBS buffer and transferred to a YPD medium containing 25 µM CuSO4 and 1 mM ascorbic acid to repress the transcription of *ZBP1*:*HA*. Yeast cells were harvested after incubation of 0, 1, 2, 4, and 6 h, respectively, and the abundance of the Zbp1:HA protein was detected by Western blotting. The results of our protein stability assay showed that the Zbp1:HA protein was degraded in a time-dependent manner over the period examined (0 to 6 h) in the wild-type background, but it was relatively stable in the *fbp1*Δ mutant, indicating that the stability of Zbp1:HA depends on Fbp1 (**Figures 2D, E**).

Next, we extracted and purified the Zbp1-HA proteins from the wild-type or *fbp1*Δ mutant strains with HA antibody and detected the polyubiquitination of Zbp1-HA by immunoblotting with ubiquitin antibody. As shown in **Figure 2F**, deletion of *FBP1* resulted in the decreased polyubiquitination of Zbp1 in *fbp1*Δ mutant compared with that in the wild-type strain, indicating that the normal polyubiquitination of Zbp1 is dependent on the function of Fbp1 in *C. neoformans*.

### Zbp1 Protein Is Localized in the Nucleus of Cryptococcal Cells

**Figure 3:**
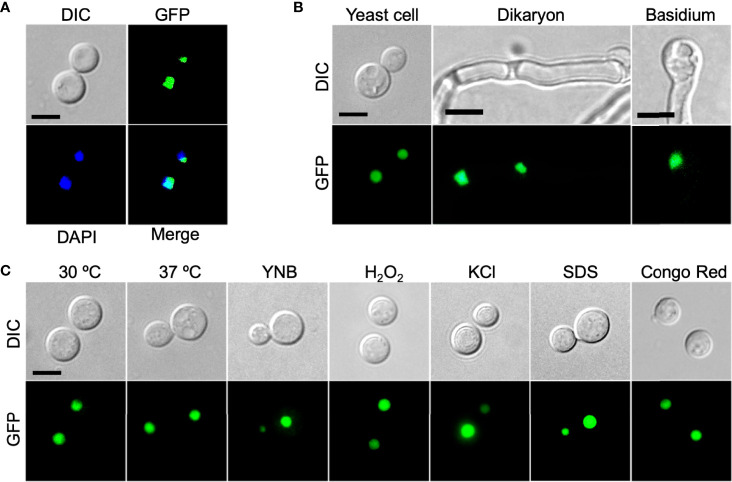
Zbp1 protein is localized in the nuclei of cryptococcal cells. **(A)** The yeast cells of the cryptococcal strain expressing the GFP-Zbp1 were observed by confocal microscopy (Olympus, FV1200). The nuclei of the yeast cells of the GFP-Zbp1 strain were also stained with 2 µg/mL DAPI solution. Scale bar, 5 μm. **(B)** The yeast cells, dikaryon, and basidium of the strain expressing GFP-Zbp1 were observed by confocal microscopy. Scale bar, 5 μm. **(C)** The localization of GFP-Zbp1 in yeast cells under different stresses. Scale bar, 5 μm.

To determine the subcellular localization of the Zbp1protein in *C. neoformans*, we made the *GFP-ZBP1* construct (pTBL187) and introduced it into both α and **a** mating type *zbp1*Δ mutant strains. The GFP-Zfp1 fusion protein was found concentrated in the nucleus of cryptococcal cells (**Figure 3A**). To further confirm the validity of the nucleus localization of GFP-Zbp1, we also examined the localization of GFP-Zbp1 in different developmental stages in *C. neoformans*. Fluorescence microscopy showed that the GFP-Zbp1 protein was also located in the nuclei of mating hyphae and basidia (**Figure 3B**).

In addition, we also detected the localization of GFP-Zbp1 fusion protein inside the yeast cell under different stress conditions such as high-temperature stress (37°C), oxidative stress (2.5 mM H_2_O_2_), osmotic stress (1.5M NaCl), and cell wall stress (0.025% SDS and 0.5% Congo red). We found that the GFP-Zbp1 protein was located in the nucleus of *Cryptococcus* cells under the above stress conditions (**Figure 3C**).

### Zbp1 Regulates Capsule Formation in *C. neoformans*

**Figure 4:**
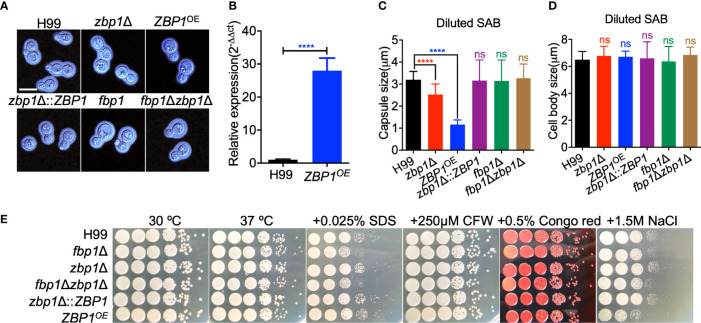
Zbp1 regulates capsule formation in *C. neoformans*. **(A)** Capsule formation was assayed in a diluted SAB medium. The yeast cells of each cryptococcal strain were grown in diluted SAB medium at 37°C for 24 hours, and the capsule formation was visualized by India ink staining. Bars, 5 µm. **(B)** The overexpression of *ZBP1* was measured by relative qRT-PCR analysis. The expression levels of the *ZBP1* gene in the wild-type strain H99 grown on YPD were set as 1, and the expression of the *GAPDH* gene was used as an internal control. ****P < 0.0001. **(C)** Statistical analysis of capsule production of each cryptococcal strain in diluted SAB medium. The capsule size from at least 100 cells was measured, and the data shown are the average with standard deviation from three repeats. ns: not significant; ****P < 0.0001. **(D)** Statistical analysis of cell body size of each cryptococcal strain in diluted SAB medium. The cell body size from the same cells in **(C)** was measured, and the data shown are the average with standard deviation from three repeats. ns, not significant. **(E)** Growth of the cryptococcal strains under different stress conditions. Overnight cultures of each strain were first diluted to an OD_600_ value of 2.0, followed by a series of ten-fold dilutions. 5 μL of each dilution were dropped on YPD plates with different stresses and incubated at 30°C for 2 days. The cryptococcal strains are shown on the left, and the culture conditions are at the top.

To evaluate the function of Zbp1 in *C. neoformans*, we generated the *ZBP1* gene deletion mutants *zbp1*Δ (TBL277 and TBL299, see **Table S1** for strain information) in both H99 and KN99**a** strain backgrounds (**Figure S1**). The *ZBP1* complemented strain *zbp1*Δ::*ZBP1* (TBL450 and TBL451), *fbp1*Δ *zbp1*Δ double mutants (TBL375 and TBL379) or *ZBP1* overexpressed strains *ZBP1* ^OE^ (TBL303and TBL296) were also constructed. The overexpression of *ZBP1* was confirmed by qRT-PCR (**Figure 4B**).

To analyze the role of Zbp1 in the virulence of *C. neoformans*, we examined the virulence factor production *in vitro* in *zbp1*Δ mutants and *ZBP1* ^OE^ strains. Compared with the wild-type H99 strain, both deletion and overexpression of the *ZBP1* gene resulted in a significant decrease in capsule production in *C. neoformans* in diluted SAB medium (**Figures 4A, C**). The average relative size of the capsule produced by the *zbp1*Δ mutant or the *ZFP1* ^OE^ cells was reduced by 46% and 51%, respectively, compared with the wild-type strain H99 (**Figure 4C**). This reduction is statistically significant (*P* < 0.0001) based on a Student’s t-test. Meanwhile, we also measured the cell body size of the yeast cells of each cryptococcal strain in **Figure 4D**, and the results showed that the average cell size of each strain was no different from that of the wild-type strain, indicating that Zbp1 is important for the capsule production in *C. neoformans*. We also examined the capsule formation of each cryptococcal strain in other capsule induction media such as MM with mannitol and DMEM with 5% CO_2_ and found that the capsule formation was similar to that of the diluted SAB, which further indicated that Zbp1 is important for the capsule production in *C. neoformans* (**Figure S2**). We then examined the growth of the *zbp1*Δ mutants and the *ZFP1* ^OE^ strains under stress conditions, and the results showed that the *ZFP1* ^OE^ strains were sensitive to 1.5 M NaCl, but not cell integrity-targeting chemicals, such as SDS, CFW, and Congo red, indicating that Zbp1 may play a role in maintaining the osmotic pressure of *Cryptococcus* cells (**Figure 4E**). Since Zbp1 may be a zinc ion binding protein that might be involved in regulating zinc or iron homeostasis, we next examined the growth of *zbp1*Δ mutant and *ZBP1* ^OE^ strain under zinc- or iron-limiting conditions. Compared with the wild-type strain H99, the *zbp1*Δ mutant and *ZBP1* ^OE^ strain showed no growth defect on the YNB medium containing 100 µM bathophenanthroline disulfonate (BPS) or 10 µM TPEN (**Figure S3**), indicating that zinc or iron deprivation did not affect the growth of these strains on YNB media.

### Zbp1 Regulates Virulence of *C. neoformans*

**Figure 5:**
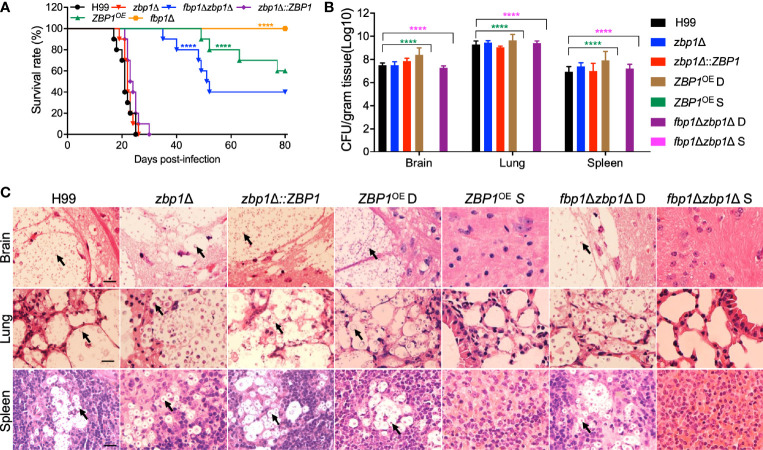
Zbp1 regulates the virulence of *C*. *neoformans* in a systematic infection model. **(A)** Survival rates of C57 BL/6 mice after infection. Female C57 BL/6 mice (ten mice per group) were intranasally infected with 10^5^ cells of each cryptococcal strain as indicated in **(A)**. Both the *zbp1*Δ mutant and the *ZBP1* ^OE^ strain are less virulent than the wild-type strain H99. ****P < 0.0001 (determined by the log-rank (Mantel-Cox) test). **(B)** Fungal burdens in the brains, lungs, and spleens of the mice at the end time point of infection. D: *ZBP1* ^OE^ or *fbp1*Δ*zbp1*Δ double mutant infected mice that died before 80 dpi; S: *ZBP1* ^OE^ or *fbp1*Δ*zbp1*Δ double mutant infected mice that survived at 80 dpi. Each data point is shown as the mean ± standard error of the mean for the values of five mice. ****P < 0.0001 (determined by Mann-Whitney test). **(C)** Hematoxylin-eosin (HE) stained slides from the cross-sections of the organs at the end time point of infection were prepared and visualized under a light microscope. The cryptococcal cells are indicated by arrows. Bars, 20 µm.

The involvement of Zbp1 in capsule production prompted us to examine its possible role in fungal virulence using a mouse inhalation model of systemic infection of *C. neoformans*. Female C57 BL/6 mice were inoculated intranasally with 1×10^5^ yeast cells, and the mice were monitored twice a day. All the mice infected with the wild-type H99 strain survived between 20 to 25 dpi, which is consistent with our previous results (Fan et al., 2019). However, disruption of the *ZBP1* gene did not affect the virulence of *C. neoformans*, as the mice infected with the *zbp1*Δ mutants survived between 19 to 26 dpi, suggesting there was no difference in virulence between the *zbp1*Δ mutant and the wild-type strain. Interestingly, the *ZBP1* ^OE^ strain showed a significant virulence attenuation compared with that of wild-type control (*P* < 0.0001, log-rank (Mantel-Cox) test), and four mice infected by the *ZBP1* ^OE^ mutant survived between 40 to 77 dpi (**Figure 5A**). However, the remaining six mice infected by the *ZBP1* ^OE^ mutant stayed healthy and continued to gain body weight during the process of the animal study, and we terminated the animal study and sacrificed the mice at 80 dpi (**Figure 5A**). In this animal study, we also set up the virulence assay of the *fbp1*Δ mutant as a control, and all the mice infected with the *fbp1*Δ mutant are survived when we terminated the animal study (80 dpi), which is consistent with our previous results (Liu and Xue, 2014; Masso-Silva et al., 2018). To test whether a *zbp1*Δ mutation can partially rescue the hypovirulence of the *fbp1*Δ mutant, we generated an *fbp1*Δ *zbp1*Δ double mutant. As expected, mice infected by the *fbp1*Δ *zbp1*Δ double mutant developed lethal infections by 40 dpi, and four mice infected by the *fbp1*Δ *zbp1*Δ double mutant still survived when we terminated the animal study (80 dpi) (**Figure 5A**), indicating that the *zbp1*Δ mutation indeed partially rescued the virulence attenuation of the *fbp1*Δ mutant.

To better understand why the *ZBP1* ^OE^ strain has a virulence defect, we examined the fungal burdens in tissues of the infected mice at the endpoint of the infection experiments. Brains, lungs, and spleens from at least three mice infected by each cryptococcal strain were collected, and the fungal burden of these tissues was evaluated as yeast colony-forming unit (CFU) per gram fresh tissue. The CFU counts showed that 10^7^, 10^9^, and 10^7^ CFU were recovered from mouse brain, lung, and spleen, respectively, when the mice were infected by the H99 strain and sacrificed at the endpoint of the experiment. Comparable CFU counts were also recovered from the tissues of the mice infected by the *ZBP1* ^OE^ strain and died before 80 dpi (*ZBP1* ^OE^ D in **Figure 5B**). Interestingly, no yeast cells were recovered from tissues of mice infected with the *ZBP1* ^OE^ strain that were still alive (*ZBP1* ^OE^ S in **Figure 5B**) when we terminated the experiment (80 dpi). We also examined the fungal burdens in tissues of the mice infected with the *fbp1*Δ *zbp1*Δ double mutant, and the CFU counts were similar to those of mice infected with the *ZBP1* ^OE^ strain (**Figure 5B**).

Meanwhile, the fungal lesion development in tissues of the *Cryptococcus*-infected mice was also examined under a light microscope in H&E-stained slides. As shown in **Figure 5C**, both the wild-type H99 and the *zbp1*Δ mutant caused severe damage in infected brains, lungs, and spleens at 23 dpi, with many yeast cells having thickened capsules. For mice infected by the *ZBP1* ^OE^ strain or the *fbp1*Δ *zbp1*Δ double mutant that died before 80 dpi, it took more than 40 days for them to cause similar damage, suggesting that the virulence of the *ZBP1* ^OE^ strain or the *fbp1*Δ *zbp1*Δ double mutant was attenuated significantly (**Figure 5C**). However, for mice that survived at 80 dpi, no detectable damage or lesion was detected in brains, lungs, or spleens infected by the *ZBP1* ^OE^ strain or the *fbp1*Δ *zbp1*Δ double mutant (**Figure 5C**). These results indicate that Zbp1 regulates the virulence of *Cryptococcus* in a murine inhalation model of cryptococcosis.

**Figure 6:**
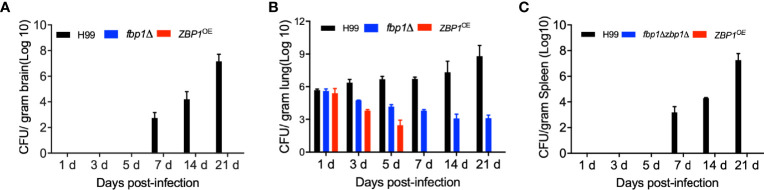
Fungal burdens in mice infected with the *ZBP1* ^OE^ strain. C57 BL/6 mice were intranasally infected with 1 × 10^5^ of the wild type, *fbp1*Δ mutant, or *ZBP1* ^OE^ strain and sacrificed at the indicated times after infection. Brains **(A)**, lungs **(B)**, and spleens **(C)** were dissected and homogenized for CFU determinations. Each data point is shown as the mean ± standard error of the mean for the values of five mice.

To better understand the virulence attenuation of the *ZBP1* ^OE^ strain, we investigated the disease progression by examining the fungal burdens of infected tissues in a time-course study. Mouse brains, lungs, and spleens by each cryptococcal strain were isolated at 1, 3, 5, 7, 14, and 21 dpi. Our results showed that no yeast cells were recovered from the brains and spleens infected by the *ZBP1* ^OE^ strain, which is the same as in the brains and spleens infected by the *fbp1*Δ mutant (**Figures 6A, C**). As a control, the fungal cells recovered from the *fbp1*Δ mutant-infected lungs remained at a persistently low level (~10^3^ CFU/g fresh lung) throughout the infection process, consistent with our previous report (**Figure 6B**) (Liu and Xue, 2014). Interestingly, the fungal burdens recovered from the lungs infected with the *ZBP1* ^OE^ strain decreased gradually with the extension of time after infection and could not be recovered after 7 dpi (**Figure 6B**).

**Figure 7:**
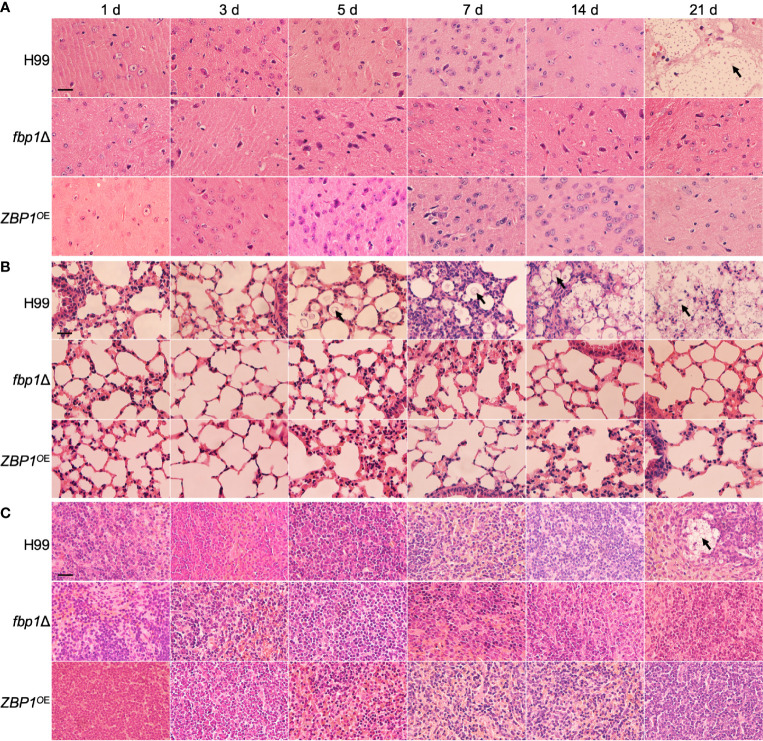
Hematoxylin-eosin (HE) stained slides from the cross-sections of the brains **(A)**, lungs **(B)**, and spleens **(C)** of the mice in **Figure 6** at different time points of infection were prepared and visualized under a light microscope. The cryptococcal cells are indicated by arrows. Bars, 20 µm.

Histopathology results of the time-course study showed that both the *ZBP1* ^OE^ strain and the *fbp1*Δ mutant could not cause infections in brains (**Figure 7A**), lungs (**Figure 7B**), and spleens (**Figure 7C**) of the infected mice when compared with the wild type-infected mice. Observation of H&E-stained slides also showed the intensive accumulation of yeast cells in tissues of the wild type-infected mice at 21 dpi, while no yeast cell could be observed from the slides of the *ZBP1* ^OE^ strain and the *fbp1*Δ mutant (**Figures 7A–C**). These results once again suggest that the tight regulation of the Zbp1 protein level is closely related to the virulence of *C. neoformans*.

### Zbp1 Is Important in Fungal Survival in Macrophages or Host Complement System

**Figure 8:**
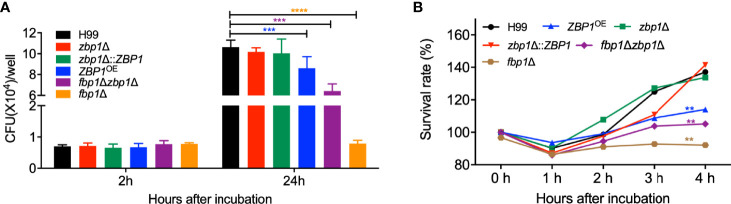
Zbp1 is important for the interaction of *Cryptococcus* with macrophage and survival in the host complement systems. **(A)** The intracellular proliferation of *C*. *neoformans* was measured using J774 macrophages. The nonadherent extracellular yeast cells were removed and phagocytosed yeasts were co-cultured for indicated times, as shown in **(A)**. The lysates were plated on YPD plates, and the CFU numbers recovered from the macrophage culture were used to determine intracellular proliferation and macrophage killing. ***P < 0.001; ****P < 0.0001 (determined by Mann-Whitney test). **(B)** The survival rate of the cryptococcal strains in **(A)** after co-incubation with fetal bovine serum. The survival rate was determined by the numbers of CFU recovered from the cultures after co-incubated with fetal bovine serum at the indicated times. The survival rate of the wild-type H99 strain was used as a control. **P < 0.01.

To better understand how Fbp1 may regulate Zbp1 function *in vivo*, we also examined the survival of *ZBP1* ^OE^ strain in macrophages or the host complement system. Our results showed that the CFU counts from the *ZBP1* ^OE^-coincubated macrophages after 2 h of incubation were comparable to that of the *fbp1*Δ mutant or the wild-type strains, indicating a similar phagocytosis level between each *Cryptococcus* strain. However, after 24 h of incubation, both the *ZBP1* ^OE^ strain and the *fbp1*Δ mutant showed reduced intracellular growth, with the *ZBP1* ^OE^ strain showing a lesser defect than the *fbp1*Δ mutant (*P* < 0.001, **Figure 8A**). The *fbp1*Δ *zbp1*Δ double mutant also showed a significant intracellular growth defect, but it was much less severe than that of the *fbp1*Δ mutant, which is consistent with the conclusion that Zbp1 is a downstream target of Fbp1.

The viability assay of the *ZBP1* ^OE^ strain after incubation with mouse serum showed that the survival rate of the *ZBP1* ^OE^ strain and the *fbp1*Δ *zbp1*Δ double mutant was significantly lower than that of the wild type after 4 h incubation with the FBS (*P* < 0.01, **Figure 8B**), indicating that components of the host complement system did have more severe damage on the *ZBP1* ^OE^ strain or the *fbp1*Δ *zbp1*Δ double mutant. However, the survival rate of the *fbp1*Δ *zbp1*Δ double mutant in mouse serum was much higher than that of the *fbp1*Δ mutant (*P* < 0.01), which also suggested that Zbp1 is a downstream target of Fbp1.

The above results suggested that the *ZBP1* ^OE^ strain proliferates slower than the wild type once it is engulfed by macrophages or survives less than the wild type in the host complement system, which could be reasons why the *ZBP1* ^OE^ strain has a significant virulence attenuation in the mouse systemic infection model.

## Discussion

F-box proteins are the key components of the SCF E3 ubiquitin ligases and are responsible for specific recognition of downstream substrates in the UPS-mediated ubiquitination degradation process. In our previous studies, we identified an F-box protein Fbp1 and revealed that it controls the virulence of *C. neoformans* without affecting classical virulence factors (e.g., melanin, capsule production, and thermal tolerance) (Liu et al., 2011b). In the process of exploring the mechanism of Fbp1 regulating the virulence of *C. neoformans*, we identified a downstream substrate protein, Isc1, and found that both the *isc1*Δ mutant and the *ISC1* overexpression strain significantly reduced fungal virulence (Liu and Xue, 2014). However, the virulence attenuation of either strain was not as severe as that of the *fbp1*Δ mutant, suggesting that Isc1may not be a direct regulator of virulence of *C. neoformans* and Fbp1 likely has additional substrates involved in the regulation of fungal virulence.

In this study, to understand how the SCF(Fbp1) E3 ligase regulates the virulence of *C. neoformans*, we further analyzed the potential Fbp1 substrates using an iTRAQ-based proteomic approach and identified the zinc-binding protein Zbp1 as a substrate of Fbp1. We showed that Zbp1 directly interacts with Fbp1 and that the ubiquitination and degradation of Zbp1 are dependent on Fbp1. We observed that the Zbp1 protein was localized in the nucleus of *C. neoformans* cells. In addition, both deletion and overexpression of the *ZBP1* gene led to reduced capsule size, while overexpression has a more significant impact on capsule size reduction in *C. neoformans*. Fungal virulence analysis revealed that disruption of the *ZBP1* gene did not affect the virulence of *C. neoformans*, but virulence was significantly attenuated in the *ZBP1* overexpression strains. These data suggested that the SCF(Fbp1) E3 ligase-mediated UPS pathway regulates the virulence of *C. neoformans* by regulating Zbp1, a zinc-binding protein.

Zinc is a micronutrient essential for the growth of all microorganisms and plays an important role in various biochemical processes, cell growth, and development (Amich and Calera, 2014). Therefore, the competition for micronutrients between pathogens and their hosts is a vital determinant in the process of infection. In this study, we identified a putative zinc-binding protein in the downstream substrate identification of F-box protein Fbp1. We first analyzed the protein sequence, but we could not find any expected zinc-binding domains such as the zinc finger domain, a specific domain of zinc-binding proteins. However, when we examined the induction effect of zinc on *ZBP1* gene expression, we found that the addition of 10 µm zinc in YPD medium resulted in a significant increase in *ZBP1* expression (**Figure 1**, *P* < 0.0001), indicating that Zbp1 may be a zinc-binding protein without a typical zinc ion binding domain. However, when we tested the growth of the *zbp1*Δ mutant and *ZBP1* ^OE^ strain under zinc- or iron-limiting conditions, the *zbp1*Δ mutant, and *ZBP1* ^OE^ strain showed no growth defect compared with the wild-type strain H99. These results indicated that zinc or iron deprivation did not affect the growth of *zbp1*Δ mutant and *ZBP1* ^OE^ strains, but the additional zinc ion can induce the expression of the *ZBP1* gene.

Zinc-binding proteins have shown to be involved in the virulence of human fungal pathogens, such as Zap1 in *C. gattii* and Zip1, Zfp1 in *C. neoformans* (Schneider Rde et al., 2012; Do et al., 2016; Fan et al., 2019), the zinc-finger protein Csr1 in *Candida albicans* and *Candida dubliniensis* (Kim et al., 2008; Bottcher et al., 2015), and zinc transporters ZrfC in *Aspergillus fumigatus* (Amich et al., 2014). Interestingly, in our studies on identifying the downstream substrates of the F-box protein Fbp1, we found that the zinc-binding protein Zbp1 is a downstream target of Fbp1, and its ubiquitination and degradation depend on Fbp1 function. Because of this, we decided to analyze the role of Zbp1 in the development and virulence of *C. neoformans*.

We first examined the role of Zbp1 in virulence factor production in *C. neoformans* and found that both disruption and overexpression of *ZBP1* lead to the reduction of capsule size, but overexpression has a more significant effect on the reduction of capsule size. In our previous study, overexpression of the *ZFP1*, a C_2_H_2_ zinc finger protein-encoding gene, exhibited reduced capsule size (~80%) in *C. neoformans* (Fan et al., 2019). These results seem to suggest that the zinc-binding proteins can regulate capsule production in *C. neoformans*. As a substrate of Fbp1, Zbp1 should be stabilized and enriched in an *fbp1*Δ mutant background. However, there was no difference in capsule formation between the *fbp1*Δ mutant and the wild-type strain (Liu et al., 2011b; Masso-Silva et al., 2018). It has also been reported that disruption of the zinc-binding proteins encoding genes (e.g., *ZIP1*and *ZIP2* in *C. neoformans* and *C. gattii*, *ZAP1* in *C. gattii*) does not affect the capsule formation in *Cryptococcus* species (Schneider Rde et al., 2012; Schneider et al., 2015; Do et al., 2016). Thus, the difference in capsule production between the *fbp1*Δ mutant and the *ZBP1* overexpressed strain seems to suggest that the capsule size reduction of the *ZBP1* overexpressed strain is caused by the Zbp1 protein itself, not the Fbp1. It is also possible that Zbp1 may be regulated by other regulatory systems besides the SCF(Fbp1) E3 ligase. As for how the zinc-binding protein Zbp1 regulates the capsule formation in *Cryptococcus* is still unknown and needs further study.

Zinc-binding proteins have been shown to be involved in the virulence of human fungal pathogens. The protein level of Zbp1 should be tightly regulated by the SCF(Fbp1) E3 ligase, and too high or too low levels of this protein may lead to functional defects and cause virulence attenuation. However, our results show no virulence attenuation in the *zbp1*Δ mutant while the *ZBP1* overexpression strain significantly reduced fungal virulence, although the virulence attenuation of the *ZBP1* overexpression strain was not as severe as that of the *fbp1*Δ mutant. The reduced fungal virulence in the *ZBP1* overexpression strain was expected since the Zbp1 protein should be stabilized and enriched in an *fbp1*Δ mutant background. However, in our study, the *zbp1*Δ mutant showed full virulence as the wild-type strain in a murine inhalation model of cryptococcosis; this may be because there may be other zinc-binding proteins in *Cryptococcus*, and one or some of the zinc-binding proteins compensate for the defects in the zinc ion binding caused by the disruption of the *ZBP1* gene. The *fbp1*Δ mutant has a more significant virulence attenuation than the *ZBP1* overexpression strain, suggesting that Fbp1 likely has additional substrates involved in the regulation of fungal virulence.

In summary, our study identified a number of potential substrates of the SCF(Fbp1) E3 ligase in *C. neoformans* and demonstrated a novel fungal virulence regulatory pathway in *C. neoformans*, involving the UPS and its regulation of zinc-binding proteins. Given the presence of other potential substrates, it would be interesting to investigate the functions of additional Fbp1 substrates.

## Data Availability Statement

The original contributions presented in the study are included in the article/**Supplementary Material**. Further inquiries can be directed to the corresponding author.

## Ethics Statement

The animal studies conducted at Southwest University were in full compliance with “Guidelines on Ethical Treatment of Experimental Animals (2006, No. 398)” issued by the Ministry of Science and Technology of China and the “Regulation on the Management of Experimental Animals (2006, No. 195)” issued by Chongqing Municipal People’s Government. The Animal Ethics Committee of Southwest University approved all of the vertebrate studies.

## Author Contributions

T-BL conceived and designed the experiments, and wrote the manuscript. L-TH and Y-JW performed the experiments and acquired the data. T-BL and L-TH Analyze the data. T-BL obtained the funding. All authors contributed to the article and approved the submitted version.

## Funding

This work was supported by the National Natural Science Foundation of China (31970145 and 32170203) and the Natural Science Foundation of Chongqing, China (cstc2021jcyj-msxmX1077).

## Conflict of Interest

The authors declare that the research was conducted in the absence of any commercial or financial relationships that could be construed as a potential conflict of interest.

## Publisher’s Note

All claims expressed in this article are solely those of the authors and do not necessarily represent those of their affiliated organizations, or those of the publisher, the editors and the reviewers. Any product that may be evaluated in this article, or claim that may be made by its manufacturer, is not guaranteed or endorsed by the publisher.
