# Supplementary material for: The F-Box Protein Fbp1 Regulates Virulence of Cryptococcus neoformans Through the Putative Zinc-Binding Protein Zbp1
Source: Front Cell Infect Microbiol. 2021 Dec 27;11:794661. doi: 10.3389/fcimb.2021.794661 (PMC8744115; doi:10.3389/fcimb.2021.794661)
Supplement: Supplementary file 1 [file DataSheet_1.docx]

Supplementary Figures


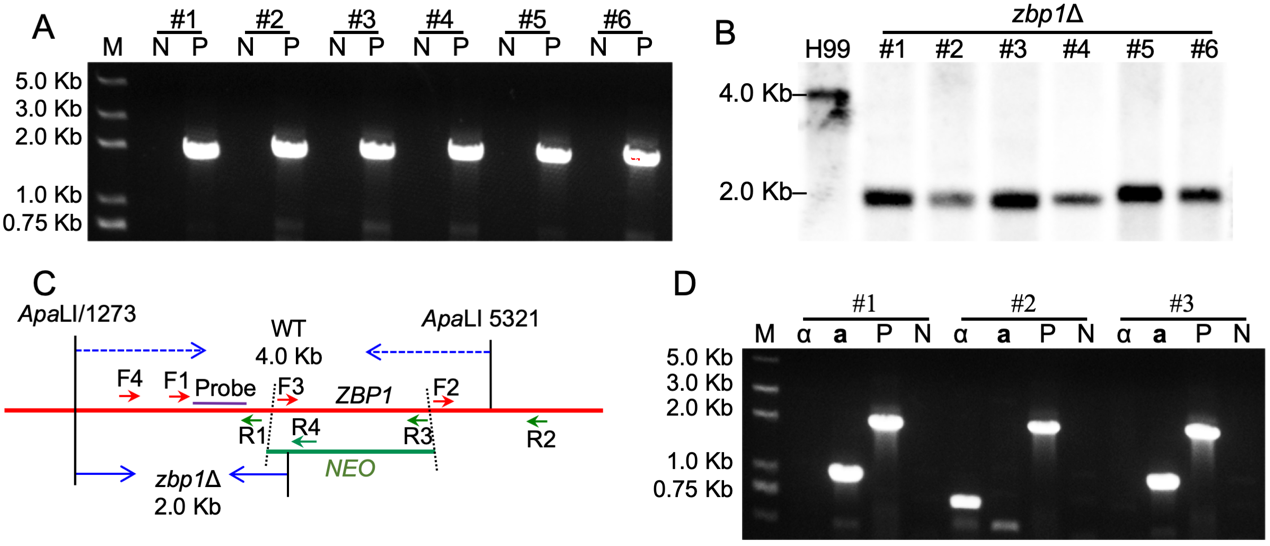


**FIGURE S1**. Generation of *zbp1*Δ mutants. (A) The diagnostic PCR validation of the six transformants with G418 resistance. P: positive primers, TL1058/TL59 (F4/R4 in S1C); N: negative primers, TL1056/TL1057 (F3/R3 in S1C). (B) Southern blotting analysis of the *ZBP1* knockout mutants. A total amount of 50 μg of each genomic DNA was digested with *ApaL*I, fractionated, and hybridized with a *ZBP1* upstream flanking sequence-specific probe, as shown in S1C. As expected, a 4.0 and 2.0 Kb bands were generated in the wild-type strain H99 and the *zbp1*∆ mutants, respectively. (C) Restriction enzyme used to digest of the genomic DNAs in Southern blotting. The probe was synthesized using the PCR products amplified with TL1052/TL1053 (F1/R1) as templates. (D) The diagnostic PCR validation of the **a** mating-type *zbp1*Δ mutants. ⍺: ⍺ mating-type specific primers, TL67/TL68; **a**: **a** mating-type specific primers, TL69/TL70. P: positive primers, TL1058/TL59 (F4/R4 in S1C); N: negative primers, TL1056/TL1057 (F3/R3 in S1C).


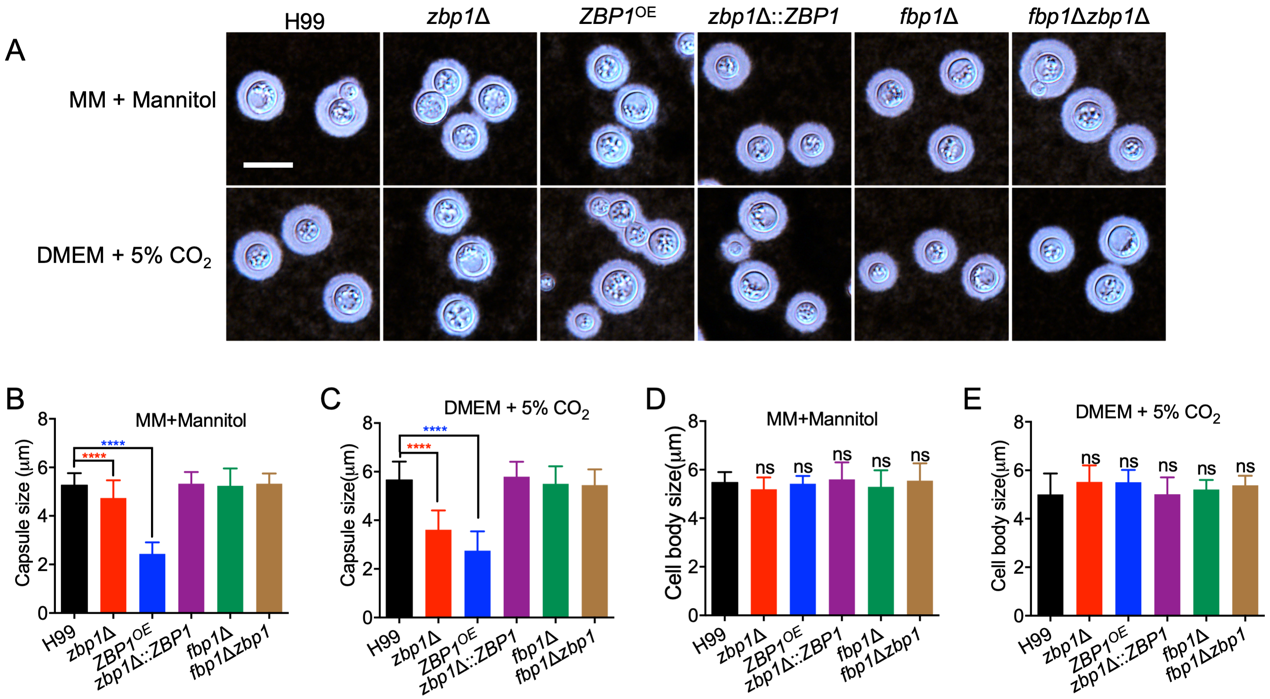


**FIGURE S2**. Zbp1 regulates capsule formation in *C. neoformans*. (A) Capsule formation was assayed in MM medium with Mannitol or DME medium with 5% CO_2_. The yeast cells of each cryptococcal strain were grown in liquid MM medium supplemented with mannitol as carbon sources at 30 ºC for 48h or on DME medium for 48 h at 30 ºC in 5% CO_2_. The capsule formation was visualized by India ink staining. Bars, 5 µm. (B) Statistical analysis of capsule production of each cryptococcal strain in MM medium with mannitol. The capsule size from at least 100 cells was measured, and the data shown are the average with standard deviation from three repeats. ****, *P* < 0.0001. (C) Statistical analysis of capsule production of each cryptococcal strain on DME medium with 5% CO_2_. The capsule size from at least 100 cells was measured, and the data shown are the average with standard deviation from three repeats. ****, *P* < 0.0001. (D) Statistical analysis of cell body size of each cryptococcal strain in MM medium with mannitol. The cell body size from the same cells in (B) was measured, and the data shown are the average with standard deviation from three repeats. ns: not significant. (E) Statistical analysis of cell body size of each cryptococcal strain on DME medium with 5% CO_2_. The cell body size from the same cells in (C) was measured, and the data shown are the average with standard deviation from three repeats. ns: not significant.


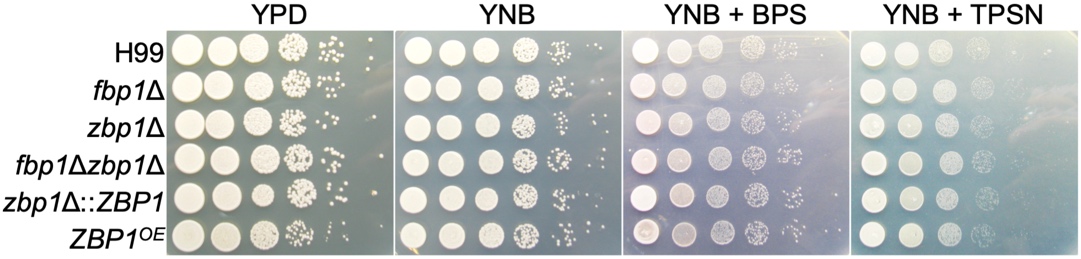


**FIGURE S3**. Growth of the cryptococcal strains under zinc- or iron-limiting conditions. Overnight cultures of each strain were first diluted to an OD600 value of 2.0, followed by a series of ten-fold dilutions. 5 μL of each dilution were dropped on YPD plates, or YNB plates supplemented with BPS or TPSN and incubated at 30 °C for 2 days. The cryptococcal strains are shown on the left, and the culture conditions are at the top.
