# Supplementary material for: The F-Box Protein Fbp1 Regulates Virulence of Cryptococcus neoformans Through the Putative Zinc-Binding Protein Zbp1
Source: Front Cell Infect Microbiol. 2021 Dec 27;11:794661. doi: 10.3389/fcimb.2021.794661 (PMC8744115; doi:10.3389/fcimb.2021.794661)
Supplement: Supplementary file 2 [file Table_1.docx]

**TABLE S1** Strains and plasmids used in this study

| Strains/plasmids | Genotype/properties | Source/reference |
| --- | --- | --- |
| *C. neoformans* | | |
| H99 | *MAT*α | (Perfect et al., 1993) |
| KN99**a** | *MAT***a** | (Nielsen et al., 2003) |
| TBL81 | *MAT*α *P_ACTIN_*-*Fbp1-Flag*::*URA5* | (Liu and Xue, 2014) |
| TBL277 | *MAT*α *zbp1*Δ::*NEO* | In this study |
| TBL296 | *MAT***a** *P_ACTIN_-Zbp1:HA*::*NAT* | In this study |
| TBL299 | *MAT***a** *zbp1*Δ::*NEO* | In this study |
| TBL303 | *MAT*α *P_ACTIN_-Zbp1:HA*::*NAT* | In this study |
| TBL311 | *MAT*α *zbp1*Δ:: *NEO* *P_H3_-GFP-Zbp1*::*NAT* | In this study |
| TBL343 | *MAT*α *fbp1*Δ:: *NEO P_CTR4_-Zbp1:HA*::*NAT* | In this study |
| TBL356 | *MAT***a** *zbp1*Δ:: *NEO* *P_H3_-GFP-Zbp1*::*NAT* | In this study |
| TBL363 | *MAT*α *P_ACTIN_*-*Fbp1*-*Flag*::*URA5 P_CTR4_-Zbp1:HA*::*NAT* | In this study |
| TBL375 | *MAT*α *fbp1*Δ::*NEO* *zbp1*Δ::*NEO* | In this study |
| TBL379 | *MAT***a** *fbp1*Δ::*NEO* *zbp1*Δ::*NEO* | In this study |
| TBL449 | *MAT*α H99:: *P_CTR4_-Zbp1:HA*::*NAT* | In this study |
| TBL450 | *MAT*α *zbp1*Δ::*NEO ZBP1*::*NAT* | In this study |
| TBL451 | *MAT***a** *zbp1*Δ::*NEO ZBP1*::*NAT* | In this study |
| *S. cerevisiae* | | |
| NMY32 | [MAT**a** his3Δ200 trp1-901 leu2-3,112 ade2 LYS2::(lexAop)_4_-HIS3 URA3::(lexAop)_8_-lacZ ade2::(lexAop)_8_-ADE2 GAL4] | DUALmembrane Kit 2 (Dualsystem, Biotech, Zürich, Switzerland) |
| YS2 | NMY32::AD-T7 BD-53 | In this study |
| YS3 | NMY32::AD-T7 BD-LAM | In this study |
| YS9 | NMY32::AD BD-Fbp1 | In this study |
| YS18 | NMY32::AD-Zbp1 BD-Fbp1 | In this study |
| YS27 | NMY32::AD BD-Zbp1 | In this study |
| YS28 | NMY32::AD-Fbp1 BD-Zbp1 | In this study |
| Plasmids | | |
| pCN19 | Amp^r^ Plasmid harboring *GFP* under histone H3 promoter | (Price et al., 2008) |
| pTBL1 | Amp^r^ Plasmid harboring *NAT* marker | (Fan et al., 2019) |
| pTBL5 | Amp^r^ Vector for *P_ACTIN_-mCherry*-*NAT* | In this study |
| pTBL151 | Amp^r^ Vector for pGADT7-Zbp1 for [yeast](javascript:;) [two-hybrid](javascript:;) | In this study |
| pTBL175 | Amp^r^ Vector for *P_ACTIN_-*Zbp1-HA-*NAT* for *ZBP1* overexpression | In this study |
| pTBL176 | Amp^r^ Vector for pGBKT7-Zbp1 for [yeast](javascript:;) [two-hybrid](javascript:;) | In this study |
| pTBL187 | Amp^r^ Vector for *P_H3_-GFP-*Zbp1-*NAT* for Zbp1 localization | In this study |
| pTBL199 | Amp^r^ Vector for *P_CTR4_-*Zbp1*-*HA-*NAT* for Zbp1 [stability](javascript:;) [assay](javascript:;) | In this study |
| pTBL215 | Amp^r^ Vector for *P_ZBP1_-ZBP1-NAT* for *ZBP1* complementation | In this study |

References

Fan, C.L., Han, L.T., Jiang, S.T., Chang, A.N., Zhou, Z.Y., and Liu, T.B. (2019). The Cys2His2 zinc finger protein Zfp1 regulates sexual reproduction and virulence in *Cryptococcus neoformans*. *Fungal Genet Biol* 124**,** 59-72.

Liu, T.B., and Xue, C. (2014). Fbp1-mediated ubiquitin-proteasome pathway controls *Cryptococcus neoformans* virulence by regulating fungal intracellular growth in macrophages. *Infect Immun* 82**,** 557-568.

Nielsen, K., Cox, G.M., Wang, P., Toffaletti, D.L., Perfect, J.R., and Heitman, J. (2003). Sexual cycle of *Cryptococcus neoformans* var. *grubii* and virulence of congenic a and alpha isolates. *Infect Immun* 71**,** 4831-4841.

Perfect, J.R., Ketabchi, N., Cox, G.M., Ingram, C.W., and Beiser, C.L. (1993). Karyotyping of *Cryptococcus neoformans* as an epidemiological tool. *J Clin Microbiol* 31**,** 3305-3309.

Price, M.S., Nichols, C.B., and Alspaugh, J.A. (2008). The Cryptococcus neoformans Rho-GDP dissociation inhibitor mediates intracellular survival and virulence. *Infect Immun* 76**,** 5729-5737.
