# Supplementary material for: The F-Box Protein Fbp1 Regulates Virulence of Cryptococcus neoformans Through the Putative Zinc-Binding Protein Zbp1
Source: Front Cell Infect Microbiol. 2021 Dec 27;11:794661. doi: 10.3389/fcimb.2021.794661 (PMC8744115; doi:10.3389/fcimb.2021.794661)
Supplement: Supplementary file 3 [file Table_2.docx]

**TABLE S2** PCR primers used in this study

| Primers | Targeted genes | Sequence (5’-3’) |
| --- | --- | --- |
| TL17 | M13F | GTAAAACGACGGCCAG |
| TL18 | M13R | CAGGAAACAGCTATGAC |
| TL19 | *NEO* split F | GGGCGCCCGGTTCTTTTTGTCA |
| TL20 | *NEO* split R | TTGGTGGTCGAATGGGCAGGTAGC |
| TL59 | *NEO* R4 | TGTGGATGCTGGCGGAGGATA |
| TL67 | *STE20A* ⍺ F | CCAAAAGCTGATGCTGTGGA |
| TL68 | *STE20A* ⍺ R | AGGACATCTATAGCAGAT |
| TL69 | *STE20A* a F | TCCACTGGCAACCCTGCGAG |
| TL70 | *STE20A* a R | ATCAGAGACAGAGGAGCAAGAC |
| TL903 | *AD-Zbp1* F | GGCATCGATACGGGATCCACATGGCTTTTAGCGACAATGACAAC(*Bam*HI) |
| TL904 | *AD-Zbp1* R | ATTCATCTGCAGCTCGAGTCATGGACCTCTCATCGCCTCT(*Pst*I) |
| TL1052 | *ZBP1* KO F1 | ACTTTTGCGATTGGCGGTCAGA |
| TL1053 | *ZBP1* KO R1 | CTGGCCGTCGTTTTACTGCTTATGCGTTTATGTTGTCGTT |
| TL1054 | *ZBP1* KO F2 | GTCATAGCTGTTTCCTGGAAGTGGAGGTATGCGGAGTTTAG |
| TL1055 | *ZBP1* KO R2 | TAGTTGCGCCTTAGCTGAGTATGT |
| TL1056 | *ZBP1* KO F3 | TTCTCGCAAGCCATCAAACTCAAC |
| TL1057 | *ZBP1* KO R3 | TGCCTCAACACCCTCCCTACATCT |
| TL1058 | *ZBP1* KO F4 | GCGGTGAATCATTTTGCTGTC |
| TL1094 | *P_Actin_-Zbp1-HA* F | CGCCCAACATGTCTGGATCCATGGCTTTTAGCGACAATGACAAC(*Bam*HI) |
| TL1095 | *P_Actin_-Zbp1-HA* R | ACGTCGTATGGGTAGGATCCTGGACCTCTCATCGCCTCTTCATA(*Bam*HI) |
| TL1096 | *BD-Zbp1* F | CCGAATTCCCGGGGATCCACATGGCTTTTAGCGACAATGACAAC(*Bam*HI) |
| TL1097 | *BD-Zbp1* R | CCGAATTCCCGGGGATCCACATGGCTTTTAGCGACAATGACAAC (*Pst*I) |
| TL1168 | GFP-ZBP1 F | GACGAGCTGTACGGATCCATGGCTTTTAGCGACAATGACAAC(*Bam*HI) |
| TL1169 | GFP-ZBP1 R | CTGGCGGCCGTTACTAGTTGGACCTCTCATCGCCTCTTCATA (*Spe*I) |
| TL1252 | *P_CTR4_-Zbp1-HA* F | TCCTGCAGCCCGGGGGATCCATGGCTTTTAGCGACAATGACAAC(*Bam*HI) |
| TL892 | *P_CTR4_-Zbp1-HA* R | ACGTCGTATGGGTAGGATCCGTAACCACCGTTGTAACCACCC(*Bam*HI) |
| TL1283 | *ZBP*1 Comp F | GATATCGAATTCCTGCAGCCCGGGGGATCCCATCTTCCCTCCTGGAGTCA  CACC(*Bam*HI) |
| TL1284 | *ZBP1* Comp R | CGGTGGCGGCCGCTCTAGAACTAGTGGATCGACTTTACCGCCCCTTATTGACTT(*Bam*HI) |
